# The Impact of the COVID-19 Pandemic on the Incidence of First Episode Psychosis in South London

**DOI:** 10.1192/bjo.2022.219

**Published:** 2022-06-20

**Authors:** Zeryab Meyer, Aryn Azlan, Edoardo Spinazzola, Diego Quattrone, Robin Murray

**Affiliations:** 1 University of Liverpool, Liverpool, United Kingdom; 2 King's College London, London, United Kingdom

## Abstract

**Aims:**

Transmission of the severe acute respiratory syndrome coronavirus-2 (SARS-CoV-2) has led to a global pandemic. Many studies are underway to ascertain the mental health impact of this seismic event, however no study has investigated its effect on psychosis incidence. We hypothesise that the overall crude incidence rates of first episode psychosis (FEP) will be higher during the pandemic when comparing the same area of South London in defined pre-pandemic and pandemic time periods.

**Methods:**

Clinical records for all patients aged 18 to 64 years presenting to early intervention in psychosis services in the London boroughs of Southwark and Lambeth between July 1st 2019 to December 31st 2019 (pre-pandemic period) and July 1st 2020 to December 31st 2020 (pandemic period) were extracted from the Clinical Record Interactive Search (CRiS), an online database containing anonymised patient records. All patients were manually screened using the Screening Schedule for Psychosis to confirm FEP, with 104/235 cases meeting criteria for FEP in the pre-pandemic period compared with 158/376 in the pandemic period. Crude, age-standardised, and sex-standardised incidence rates and ratios were calculated for interpretation.

**Results:**

The crude incidence rate of FEP was significantly higher in the pandemic period (68.3, 95% CI:[57.6 ; 78.9]) than the pre-pandemic period (44.9, 95% CI:[36.3 ; 53.6]). The crude incidence ratio was 1.52 (95% CI:[1.28 ; 1.77]), indicating that the overall crude incidence of FEP in the pandemic period was significantly higher (52%) than in the pre-pandemic period. The directionality and statistical significance of this ratio was unperturbed by standardisation for age (SIR = 1.45, 95% CI[1.23 ; 1.70]) and sex (SIR = 1.56, 95% CI[1.33 ; 1.83]).

**Conclusion:**

Our results suggest that the COVID-19 pandemic has had a significant impact on the incidence of FEP in the South London boroughs of Southwark and Lambeth. Further research is required to elucidate the factors contributing to this increase to inform targeted interventions and prevent deterioration in at-risk patients.

